# Consumers’ Willingness to Pay for Pharmacist Counselling Services and the Factors Affecting It in Community Pharmacies

**DOI:** 10.5812/ijpr-132736

**Published:** 2023-04-17

**Authors:** Omid Soodi, Elahe Hesari, Reza Hojjatifard, Meysam Seyedifar

**Affiliations:** 1 Department of Pharmacoeconomics and Pharmaceutical Administration, School of Pharmacy, Tehran University of Medical Sciences, Tehran, Iran; 2 Department of Epidemiology and Biostatistics, School of Public Health, Tehran University of Medical Sciences, Tehran, Iran; 3 Pharmaceutical Management and Economic Research Center, the Institute of Pharmaceutical Sciences (TIPS), Tehran University of Medical Sciences, Tehran, Iran

**Keywords:** Pharmacists, Pharmacy, Willingness to Pay (WTP), Services, Survey

## Abstract

**Background:**

Community pharmacists play an important role in improving outcome by providing advice and counselling services to patients.

**Objectives:**

The aim of this study was to measure the willingness to pay (WTP) for pharmacist counselling services in community pharmacies and identify determinant factors on consumers’ WTP.

**Methods:**

A self-administered questionnaire-based survey was conducted in community pharmacies in Tehran (capital of Iran) from January 1, 2020 to February 20, 2021. Contingent valuation method was applied to evaluate respondents’ maximum WTP using three hypothetical scenarios illustrating different levels of counselling services. Logistic regression was used to analyze the association between different variables and WTP for pharmacy services.

**Results:**

Total number of participants who completed the questionnaire were 332 and 60% of the participants were male. In the first scenario 70.2% of participants were willing to pay for oral counselling pharmacy services. In the second and third scenario, percentage of people willing to pay increased to 79.5%. and 86.1%, respectively. In the first scenario, monthly income (OR = 0.041, P value = 0.04), the duration of underlying illness (OR = 0.04, P value = 0.04) and the using internet (OR = 2.59, P value = 2.59) had a statistically significant relationship with willingness to pay. In the third scenario, the willingness to pay increased as the age decreased. The possibility of using the internet (OR = 3.32, P value = 0.00) and the need for a community pharmacist (OR = 2.19, P value = 0.03) increased the chance of willingness to pay.

**Conclusions:**

More consumers are willing to pay for more pharmacist counselling services. Therefore, improving the quality of counselling services could have positive economic effects on community pharmacies.

## 1. Background

Community pharmacists play an important role in improving the outcome of treatment and increasing patients’ quality of life (1, 2). They can provide advice and counselling services to patients along with information about medicine, such as preventable adverse events and interactions between prescription drugs. This counselling has been associated with reduction in drug errors, increasing the effectiveness and safety of the medication, and reduction in morbidity and mortality (2-5). Evidence has shown that patient counseling and receive instruction from pharmacists made fewer medication errors and prevent adverse drug events (4, 6, 7). Also, community pharmacist, along with the prescriber, is responsible for ensuring that patients are aware of the risk of side effects and take appropriate action if they occur (8). The pharmacist’s contribution to the optimization of drug therapy may be evaluated by ascertaining the number of drug-related problems addressed or prevented, or by assessing the clinical outcomes for the patients (9). Studies demonstrated that a statistically significant 66% decrease in preventable adverse drug effects (ADEs) due to medication ordering by pharmacist (10, 11). Pharmacists are particularly well positioned to provide the necessary medication instruction to patients, and also, they provide drug monitoring and corresponding feedback to assist physicians in prescribing decision making and laboratory monitoring (11-13).

Numerous studies showed that community pharmacists' services significantly increase clinical efficiency. Community pharmacists’ consultations also have economic benefits for the country's healthcare system by recommend patients to use generic medicines instead of high-priced branded medicines (14-18). Preventable adverse drug reactions in an in-patient setting, costs (per hospitalization) ranged from a minimum of $2,851 to a maximum of $9,015 based on a systemic review of observational study (19). Community pharmacists can help patients avoid drug side effects and hospitalizations from self-medication with over-the-counter (OTC) drugs (18). Pharmacists have demonstrated reducing healthcare costs by: (a) preventing hospital admission or reducing hospital length of stay (LOS); (b) changes in the expense on a patient’s treatment, e.g., switching from intravenous to oral therapy where appropriate (17).

There are various tools for economic evaluation of services. Cost benefit analysis is a widespread approach in economic evaluation. One of the most common techniques applied in cost benefit analysis is willingness to pay (19). Willingness to pay (WTP) is the maximum amount measured in monetary units, that an individual would be willing to sacrifice to receive the health care program's benefits (20, 21). The most important determinant factors effecting WTP are age, sex, marital status, job, insurance status, education, income/wealth, perceived own susceptibility, and perceived severity of disease (22). Contingent valuation is useful method to understand, for consumers how valuable is a service or a product which has underdeveloped markets. This method has been suggested to measure the economic value of community pharmacist provided services, since the market for these services is currently underdeveloped (19).

In Gore and Madhavan study in 1994, of the consumers who indicated a willingness to pay for pharmacist counselling for non-prescription medicines, 56.5% were willing to pay between 50 cents and $1.50, 28 - 2% between $1.51 and $3, and about 15 - 3% were willing to pay more than $3 (23). In a questionnaire-based survey in Serbia in 2017, of 444 respondents, 167 (38%) reported that they were willing to pay for a medication management service provided in the community pharmacy. Significant association was observed between the willingness to pay for pharmacist-provided service and respondents’ socio-demographic factors, health-related characteristics, and behavior, dilemmas, or need for certain pharmacist provided service (2).

So far, no study has been conducted on consumers’ willingness to pay for pharmacist counselling services in Iran, which highlights the need for such a study. It is also necessary to evaluate consumers’ willingness to pay determinant factors for health planning and policy in the pharmacoeconomics field in Iran.

## 2. Objectives

The aim of this study is investigating the willingness to pay of customers for pharmacist counselling services in community pharmacies and evaluate its determinant factors.

## 3. Methods

### 3.1. Study Design and Settings

This cross-sectional survey was conducted in community pharmacies in Tehran (capital of Iran) from January 1, 2020 to February 20, 2021.

The city of Tehran was divided into 4 regions: North, south, east and west, and two community pharmacies were randomly selected from each region. A total of 8 community pharmacies were selected for distribution of the questionnaire, which included both public and private pharmacies. Community pharmacies were selected from various districts to include the different economic groups living in Tehran.

### 3.2. Participants

Participants included adult male and female (aged 18 years or older) waiting at the pharmacy for their medications to be dispensed. Patients who did not consent to participate and those who returned incomplete questionnaires were excluded. Therefore, 18 patients were excluded from the study.

The process of obtaining informed consent was as follows: A text was attached at the beginning of the questionnaire and it was explained that this research is done by a group of researchers from Tehran university of medical sciences and participation in it is completely voluntary. This study is for research purposes only and will have no effect on the preparation of participants' medications or the use of pharmacy services. The questionnaires were filled out anonymously and the participants' answers remained confidential to the researchers. This study obtained approval from Ethics Committee of Tehran University of Medical Sciences (IR.TUMS.TIPS.REC.1399.086).

We used sample size calculation for cross sectional studies/surveys with a confidence level of 90% and margin of error of 5% and sample size was calculated to be 332 (24).

### 3.3. Data Collection and Questionnaire

A questionnaire designed by the authors was given to the participants. A comprehensive search in PubMed, Scopus, Embase and Web of Science databases was performed based on the keywords such as willingness to pay, pharmacy, pharmacist, services and counselling, and similar articles were found and questions were selected based on them (2, 19). After extracting the inventory list of questions, members of the focus group discussion were formed and the list of extracted questions was presented to the group for suggestions. The purpose of the focus group discussion is to add a questionnaire if a question or other item is needed, depending on the comments of the group members. Group members include community pharmacist, pharmacy staff and professors specializing in pharmacoeconomic. A checklist is provided to group members to evaluate each question in terms of relevance, clarity, and importance.

The first section of the questionnaire was designed to elicit the socioeconomic information including gender, age, education, monthly household expenses, employment status, being health care professional or not, and internet use. The second section of questionnaire included items eliciting information about respondents’ health-related characteristics and behavior including the presence of chronic disease, duration of the chronic disease, medication therapy for chronic disease, frequency of community pharmacy that patients referred to, number of community pharmacies visited on regular basis, and main source of information on medicines.

The items in the third section were designed to elicit the respondents’ medication related problems and dilemmas such as the need for written information about medication from the community pharmacist, being hesitant about continuing your medication therapy due to adverse effects, and need for further information about medication administration.

The final section of questionnaire included items eliciting the respondents’ willingness to pay for community pharmacists counselling services that the answers were yes/no.

### 3.4. Validity and Reliability

To assess the face validity, 10 patients were interviewed separately and their views on the complexity, clarity and ambiguity of the items was used to correct the questions. Also, in a 10-member panel consisting of pharmacoeconomic experts and community pharmacists, opinions were examined and applied in the questionnaire to find the level of difficulty, the degree of inconsistency, ambiguity of expressions or the existence of inadequacies in the meanings of words. Reliability was estimated 0.7 by Cronbach's alpha coefficient.

### 3.5. Willingness to Pay Scenarios

Chosen method for estimating customers’ willingness to pay was contingent valuation. Contingent valuation (CV), in which the respondent is asked to value a contingent or hypothetical market, is a direct method used to elicit the monetary values or the willingness to pay amounts. To elicit WTP values, the respondents are presented with a hypothetical market describing the benefits of a particular health care intervention (program, pharmaceutical, medical device) (25-27).

In our study, three hypothetical scenarios with various levels and attributes were set for community pharmacist’ services. In the first scenario, pharmacist’s services contained oral counselling to optimize health outcomes of the patients, such as drug administration, drug-drug interactions or drug-food interactions, possible adverse effects, and possible ways to avoid or reduce them. In the second scenario, written counselling and recommendations (the indication of prescribed drugs, how to take them, how to store them, possible interactions between prescribed drugs or drug-food interactions and the ways to reduce them, possible side effects of the prescribed drugs and how to avoid or minimize them, how long the duration of a treatment is, what to do if a dose is forgotten) were added to oral counselling. Considering that in the new regulations of the Ministry of Health and Medical Education (MOHME) of Iran, the existence of a medical consultation space is required (28), in the third scenario, in addition to oral and written counselling, a small counselling room was considered for the patients to talk with the pharmacist in privacy. For each scenario, customers’ willingness to pay was measured. The participants were asked to choose one of the answers (yes/no) for each scenario.

### 3.6. Statistical Analysis

For quantitative data, average and standard deviation and for qualitative data, frequency and percentage were used. Statistical analyzes were performed with SPSS version 25 software. The logistic regression was used to test the association between the determinant factors and WTP. Confidence interval for all the tests were considered 95% and P value < 0.05 was considered significant.

## 4. Results

### 4.1. Baseline Characteristics

Total number of participants completing the questionnaire was 332. Majority of the respondents were male and were in the age groups of 45 - 54 and 35 - 44, respectively. Most participants had higher education than high school and were employees or retired. Majority of the respondents (87%) were able to use internet (Table 1).

**Table 1.** Baseline Characteristics (Total N = 332)

Variables	Frequency (%)
**Gender **	
Male	200 (60.2)
Female	132 (39.8)
**Age**	
18 - 24	64 (19.3)
25 - 34	58 (17.5)
35 - 44	73 (22)
45 - 54	100 (30.1)
55 - 64	25 (7.5)
64 <	12 (3.6)
**Education**	
Diploma or less	85 (25.6)
Associate degree or bachelor degree	175 (52.7)
Master degree	58 (17.5)
PhD or higher	14 (4.2)
**Employment status**	
Unemployed	15 (4.5)
Employee	119 (35.8)
Manual worker	48 (14.5)
Self employed	66 (19.6)
Retired	40 (12)
Employer	10 (3)
Other	16 (4.8)
**Being healthcare professional**	
Yes	49 (14.8)
No	283 (85.2)
**Internet user**	
Yes	289 (87)
No	43 (13)
**Monthly household expenses ($)**	
< 476	31 (9.3)
476 - 714	51 (15.4)
714 - 952	72 (21.7)
952 - 1430	74 (22.3)
1430 - 2380	25 (7.5)
> 2380	79 (23.8)

### 4.2. Health-Related Characteristics

Most of the participants had no underlying disease. Majority of respondents with a chronic disease took medication for their disease. Additionally, most of the respondents reported visiting a pharmacy two or three times a week. Physicians were the respondents’ primary sources of information on medicines or medical conditions (Table 2).

**Table 2.** Health-Related Characteristics and Medication Related Problems and Dilemmas of Respondents

Variables	Frequency (%)
**Chronic disease (N = 332)**	
Yes	97 (29.2)
No	235 (70.8)
**Taking medicine for chronic disease (N = 103)**	
Yes	82 (24.7)
No	21 (6.3)
**Main source of information on medicines (N = 332)**	
Physician	199 (59.9)
Pharmacist	53 (16)
Relatives or friends	9 (2.7)
Internet	71 (21.4)
**Frequency of referred to pharmacy (N = 332)**	
Once a month or less	5 (1.5)
Once every 2 weeks	14 (4.2)
One time a week	43 (13)
Tow to 3 times a week	270 (81.3)
**Number of pharmacies visited on regular basis**	
A regular customer of a pharmacy	44 (13.3)
Customer of two to three special pharmacies	151 (45.5)
Not a regular customer of a particular pharmacy	137 (41.3)
**Need for written information by community pharmacist (N = 332)**	
Yes	281 (84.6)
No	51 (15.4)
**Being hesitant about continuing medication therapy due to adverse effects (N = 316)**	
Yes	176 (53)
No	140 (42.2)
**Need for further information about medication administration (N = 332)**	
Yes	277 (83.4)
No	55 (16.6)
**Experience of getting counselling from community pharmacists (N = 332)**	
Community pharmacists often spend enough time for counseling patients	61 (18.4)
Community pharmacists sometimes spend enough time for counseling patients	142 (42.8)
Community pharmacists seldom spend enough time for counseling patients	129 (38.9)

In addition, most of the respondents reported that they needed a written information about their medicines by a community pharmacist when they went to a community pharmacy and stated that they needed more information about their medication. Only 19% of the respondents stated that most community pharmacies provide adequate advice and information for them (Table 2).

### 4.3. Willingness to Pay

The majority of participants were willing to pay for pharmacy services. The participants' willingness to pay for the third scenario (i.e., in addition to oral and written counselling, a small counselling room was considered for the patients to talk with the community pharmacist in privacy) was the highest (Table 3).

**Table 3.** Willingness to Pay for Three Scenarios (Total N = 332)

Willingness to Pay	No. (%)
**WTP for scenario 1**	
Yes	223 (70.2)
No	99 (29.8)
**WTP for scenario 2**	
Yes	264 (79.5)
No	68 (20.5)
**WTP for scenario 3**	
Yes	286 (86.1)
No	46 (13.9)

Abbreviation: WTP, willingness to pay.

In the first scenario, monthly income, the duration of underlying illness and the using internet had a statistically significant relationship with willingness to pay. The income below $476 had a statistically significant relationship with unwillingness to pay (OR = 0.041, P value = 0.04). People whose underlying disease was more than 12 months old did not want to pay for pharmacy services (OR = 0.04, P value = 0.04). Also, people who could using internet were willing to pay for pharmacy services (OR = 2.59, P value = 2.59) (Table 4).

**Table 4.** Logistic Regression Analysis to Test the Association Between the Determinant Factors and Willingness to Pay (Total N = 332)

Variables	WTP Scenario 1			WTP Scenario 2			WTP Scenario 3		
	B	P Value	OR	B	P Value	OR	B	P Value	OR
**Sex**	0.14	0.54	1.16	0.23	0.40	1.26	0.23	0.49	1.25
**Age**									
18 - 24	1.13	0.09	3.09	0.87	0.20	2.40	2.13	0.00	8.42
25 - 34	0.71	0.27	2.04	0.87	0.21	2.40	1.83	0.01	6.19
35 - 44	0.29	0.64	1.33	0.58	0.38	1.80	1.43	0.03	4.21
45 - 54	0.41	0.50	1.51	0.63	0.33	1.80	1.56	0.01	4.78
55 - 60	0.41	0.56	1.51	0.69	0.38	2.00	1.05	0.17	2.85
60 < ^a^									
**Employment status**									
Manual worker	1.54	0.14	4.66	1.26	0.23	3.55	0.63	0.55	1.89
Employee	-0.55	0.86	0.94	0.22	0.53	1.25	0.25	0.54	1.32
Self employed	-0.8	0.02	0.41	-0.36	0.40	0.69	-0.61	0.22	0.54
Retired	-0.57	0.11	0.56	-0.31	0.42	0.72	-0.67	0.14	0.50
Others ^a^									
**Education**									
Diploma	-1.02	0.19	0.35	-0.61	0.44	0.54	-0.54	0.49	0.57
Associate and bachelor's degree	-0.95	0.22	0.38	-0.35	0.65	0.70	0.35	0.65	1.42
Master’s degree	-0.82	0.32	0.43	-0.44	0.58	0.63	0.36	0.67	1.44
PhD degree and higher ^a^									
**Health related job**	-0.04	0.89	0.99	0.91	0.06	2.50	0.38	0.44	1.46
**Monthly household expenses ($)**									
< 476	-0.88	0.04	0.41	-0.40	0.38	0.66	-1.27	0.02	0.28
476 - 714	0.02	0.96	1.02	0.53	0.25	1.70	-0.79	0.14	0.45
714 - 952	-0.13	0.71	0.87	0.36	0.26	1.43	-0.50	0.33	0.60
952 - 1430	-0.10	0.77	0.90	0.42	0.30	1.52	-0.28	0.60	0.75
1430 - 2380	0.70	0.24	2.02	0.23	0.67	1.26	-0.33	0.64	0.71
> 2380 ^a^									
**Chronic disease**	0.24	0.05	1.27	0.10	0.48	1.10	0.22	0.18	1.24
**The duration of the disease**									
Under 12 months	0.66	0.42	1.95	0.07	0.93	1.07	0.75	0.49	2.12
Above 12month	-1.68	0.04	0.04	-0.08	0.92	0.91	-0.22	0.79	0.79
**Taking medication**	0.61	0.21	1.85	0.10	0.85	1.11	0.66	0.24	1.94
**Number of** **pharmacies visited on regular basis**									
1 pharmacy	0.13	0.73	1.13	-0.19	0.66	0.82	0.02	096	1.02
2 to 3 pharmacies	-0.07	0.78	0.92	-0.28	0.35	0.75	0.11	0.76	1.11
Not a regular customer of a particular pharmacy ^a^									
**Using Internet**	0.95	0.00	2.59	0.73	0.04	2.07	1.20	0.00	3.32
**Priority information**									
Physician	0.03	0.90	1.03	0.11	0.73	1.12	-0.20	0.62	0.81
Pharmacist	0.74	0.10	2.07	0.71	0.17	2.03	0.46	0.45	1.59
Friends	-0.04	0.95	0.95	-0.54	0.47	0.58	-0.67	0.44	0.50
Internet ^a^									
**Need a community pharmacist**	0.38	0.23	1.46	0.36	0.30	1.44	0.78	0.03	2.19
**Side effects**	0.42	0.08	1.52	0.40	0.15	1.50	0.37	0.26	1.45
**Need for information**	0.12	0.68	1.13	0.33	0.33	1.39	0.24	0.55	1.27
**Community pharmacist spend enough time to guide the clients**									
Mostly	-0.04	0.88	0.95	0.56	0.17	1.75	-0.97	0.02	0.37
Sometimes	0.43	0.11	1.54	0.20	0.50	1.22	-0.44	0.26	0.64
Rarely ^a^									

Abbreviations: WTP, willingness to pay; OR, odds ratio; B, the beta values, or b coefficients.

^a^ Reference cases, for options with yes/no answer, no is the reference case.

In the second scenario, only the using internet had a statistically significant relationship with the willingness to pay (OR = 2.07, P value = 0.04) (Table 4).

In the third scenario, the willingness to pay increased as the age decreased. The possibility of using the internet (OR = 3.32, P value = 0.00) and the need for a community pharmacist (OR = 2.19, P value = 0.03) increased the chance of willingness to pay. As in the first scenario, the monthly income below $476 had the lowest chance of willingness to pay for the community pharmacy service (OR = 0.28, P value = 0.02). In the third scenario, if the community pharmacist spent enough time to guide the clients, a statistically significant relationship with the willingness to pay was observed (OR = 0.037, P value = 0.02) (Table 4).

## 5. Discussion

In the first scenario 70.2% of participants were willing to pay for oral counselling pharmacy services. In the second scenario, written medical information about patients’ prescription drugs added to oral counselling services and the percentage of people willing to pay increased to 79.5%. This is implying that obtaining written information was a valuable service for the respondents. In the third scenario, providing counselling room for patients added to previous services. The percentage of people willing to pay increased to 86.1% which is the highest amount among the three scenarios. Therefore, counselling room which is not available in most community pharmacies in Iran is a valuable option from respondents’ perspective. Our results showed that the higher the levels of community pharmacy services, the more consumers are willing to pay for community pharmacist services.

In the first scenario, monthly household expense, the duration of underlying illness and the using internet had a statistically significant relationship with willingness to pay. In the second scenario, only using internet had a statistically significant relationship with the willingness to pay.

Finally, in the third scenario, the willingness to pay increased as the age decreased. The possibility of using the internet and the need for a community pharmacist increased the chance of willingness to pay. As in the first scenario, the monthly household expenses below $476 had the lowest chance of willingness to pay for the pharmacy service.

Our findings are partly similar to that of previous studies. Studies have revealed that for less complex pharmacy services, such as dispensing, patients were willing to pay much smaller amounts of money in comparison to more specialized services, such as medication therapy management (29, 30). Therefore improving the level of community pharmacist services could have positive economic effects on pharmacies and providing counselling services with higher quality for customers would bring more revenue for pharmacies. Previous studies have also demonstrated that community pharmacist counselling has a positive effect on patients’ drug and therapy knowledge, therapy adherence, quality of life, and reduction of medication-related problems (31, 32).

In this study, willingness to pay for pharmacy service was correlated with age and monthly household expenses while education did not correlate with willingness to pay for community pharmacist service which is similar to previous findings (33-36). There was a slight negative correlation between age and willingness to pay for community pharmacist service, which is comparable to results from another study. In 2010, Friedrich et al. surveyed a convenience sample of grocery store pharmacy customers in Chicago, Illinois regarding their willingness to pay for medical therapy management services from pharmacists. Same as our results it was found that younger patients were more willing to pay for these services (37). Based on a literature review assessing the consumers’ willingness to pay for pharmacy services, there was no correlations between sex and customers’ willingness to pay in most studies (38). However, in 2008, Schuh and Droege conducted a survey among a sample of pharmacy customers in the three Florida cities of Minnesota, Jacksonville, and Clearwater. Their objective was to determine the customer's WTP out-of-pocket for cognitive services that included medical therapy management and education services using a self-administered questionnaire. The authors observed that female patients were more willing to pay for cognitive services (39). There was a slight positive correlation between manual household expenses and willingness to pay. It is probably because people with higher household expenses typically have higher income; hence, they can pay more amounts for pharmacist services. In several previous studies, income was also positively correlated with customers’ willingness to pay (39, 40). In the current study, the household expenses were demanded instead of household income since sometimes people prefer not to declare their real income. The respondents may not be aware of the household income or for some reasons the income may change in the short term, but the consumption pattern usually does not change quickly.

### 5.1. Strength and Limitations

To the best of our knowledge, the current study is the first study in Iran assessing consumers’ willingness to pay for community pharmacist counselling services in community pharmacies. Our study evaluated the willingness to pay for three different scenarios to determine how valuable it is from the consumers’ point of view to increase the level of services.

The main limitation was the small sample size and small number of pharmacies among which the questionnaires were distributed. Although pharmacies were chosen from various districts of city to include all the economic classes of the people, yet if the more pharmacies were included, the results would be more reliable. In addition, since the study was designed as cross-sectional, it was impossible to determine the causality.

### 5.2. Conclusions

In conclusion, our study showed that consumers welcome the development of high-quality community pharmacist consultation services and more people are willing to pay for more services.

Also, our study showed that consumers' willingness to pay is related to age, monthly household expenditure, using internet, need for written information by community pharmacist and experience of getting counselling from community pharmacists.

## Data Availability

The data presented in this study are uploaded during submission as a supplementary file and are openly available for readers upon request.
